# Risk Assessment of Gas Leakage from School Laboratories Based on the Bayesian Network

**DOI:** 10.3390/ijerph17020426

**Published:** 2020-01-08

**Authors:** Xiao Zhang, Xiaofeng Hu, Yiping Bai, Jiansong Wu

**Affiliations:** 1 School of Information Technology and Network Security, People’s Public Security University of China, Beijing 102628, China; 2018211242@stu.ppsuc.edu.cn; 2 School of Emergency Management and Safety Engineering, China University of Mining & Technology, Beijing 100083, China; 1410120101@student.cumtb.edu.cn (Y.B.); 201310@cumtb.edu.cn (J.W.)

**Keywords:** laboratory safety, gas leakage, risk assessment, Bayesian network

## Abstract

In recent years, concerns about the safety of laboratories have been caused by several serious accidents in school laboratories. Gas leaks in the laboratory are often difficult to detect and cause serious consequences. In this study, a comprehensive model based on the Bayesian network is established for the assessment of the gas leaks evolution process and consequences in school laboratories. The model can quantitatively evaluate the factors affecting the probability and consequences of gas leakage. The results show that a model is an effective tool for assessing the risk of gas leakage. Among the various factors, the unsafe behavior of personnel has the greatest impact on the probability of gas leakage, and the concentration of toxic and harmful gases is the main factor affecting the consequences of accidents. Since the probability distribution of each node is obtained based on the experience of experts, there is a deviation in the quantitative calculation of the probability of gas leakage and consequences, but does not affect the risk analysis. This study could quantitatively assess the probability and consequences of gas leakage in the laboratory, and identify vulnerabilities, which helps improve the safety management level of gas in the school laboratory and reducing the possibility of gas leakage posing a threat to personal safety.

## 1. Introduction

There have been more than 260 accidents in chemical laboratories in the United States, and most accidents have caused casualties since 2001, according to statistics. Most of these accidents occurred in school laboratories and should have more attention paid to them, and they need to be analyzed deeply. School laboratories have complex environments and different kinds of risks, including fires, explosions, electric shocks, leaks, etc., where the leakage of toxic and harmful gases is difficult to detect and prevent. After accidents happen, hazard identification or risk analysis are always missing. We have analyzed some cases of gas leakage accidents in laboratories in the past ten years. The details of these cases show that the causes of gas leakage are similar, and the consequences of the accident are more serious if not handled properly. In 2015, during the replacement of a gas cylinder in a laboratory of the Shanghai Jiao Tong University in China, the H_2_S in the cylinder leaked and poisoned one worker due to inhalation of H_2_S. Therefore, it is necessary to conduct risk assessments of the leakage of toxic gases in school laboratories, which is of great significance to ensure personnel safety.

In the past ten years, numerous studies have focused on s the safety of laboratories [1,2,3,4,5,6,7]. Many models and tools are used to identify laboratory hazards, such as the ‘bowtie diagram’ and ‘Assessment and Classification of Hazards in Laboratories’ (ACHiL) [8,9,10]. Some studies have studied the risks of fire and explosion in laboratories, as well as the emergency management model, hoping to minimize the possibility of casualties in the future [11]. Not only that, but some studies have used process hazard analysis (PHA) and vulnerability assessment methodology for chemical facilities (VAM-CF) methods to assess the risks of chemical facilities [12,13]. Furthermore, a large number of studies have focused on risk assessment of laboratories. Leggett described a straightforward technique designed to identify and assess the hazards of conducting a chemical synthesis in the research environment. He also discussed the relationship between the hazards and consequences of an upset event, the likelihood of the upset happening, and the resulting risk to personnel, property, and the environment [14,15]. Ouédraogo et al. proposed a new approach named laboratory assessment and risk analysis—LARA to assess risks in the research/academic environment. The core of this methodology relies on defining adequate role player factors to assess risks in the research environment and their mathematical combination to proceed quantitative risk assessment [16,17]. Research on gas leaks has focused on gas pipeline leak hazards, leak detection, monitoring indoor air quality, and compressed gas treatment [18,19,20,21,22]. However, there are few studies on the impact of personnel, equipment, management, and other factors on the probability and consequences of gas leakage in laboratories. Therefore, we attempted to analyze the factors affecting the probabilities and consequences of gas leaks in laboratories based on the Bayesian network, and construct a risk assessment model to undertake dynamic quantitative risk assessment to analyze the risk of gas leakage in school laboratories.

There is not a well-accepted definition of the concept of risk. There are many different aspects to understand and illustrate the definition of risk. Some definitions are based on probability, chance, or expectation, some are illustrations of unexpected consequences or dangers, and others rely on uncertainty. Some consider risk to be subjective and cognitive, depending on the knowledge available, while others separate the ontological state of risk from the evaluator. Aven thoroughly discussed and summarized these definitions, their principles, advantages and disadvantages, and recent development trends [23]. Not only that, Aven also put forward some novel understandings of risks [24,25]. In this study, risk is the combination of probability of an event and its consequences [26]. The assessment of the risk of gas leakage in the laboratory is mainly about the probability of gas leakage and the consequences caused by gas leakage to express uncertainty in terms of probability. Through the probability of gas leakage, and the severity of consequences obtained, the risk of gas leakage was evaluated to improve the safety management level of gas leakage in the laboratory.

There are various qualitative risk assessment methods, which are easily applied and rely more on experts instead of data and equations. Commonly used quantitative risk assessment methods, such as fault tree analysis and event tree analysis, are visualized and computationally simple, but these methods tend to ignore the causal relationship between risk factors and cannot update dynamically. When multiple risk factors are managed at the same time, it is difficult to realize the linked calculation and comparative analysis of their respective risks for multiple scenarios with common features due to the complicated evaluation operation. The complexity and dynamic characteristics of the risk of gas leakage in school laboratories have provided the possibility for Bayesian networks to be applied to the risk assessment of this scenario. Although the structure of the bow-tie diagram (BT) is clear, it cannot describe the evolution of the scenario and the results of risk analysis when multiple causes occur simultaneously. The connection between the Bayesian network and the risk of gas leakage in the laboratory is mainly reflected in three aspects: (1) When a certain factor changes, the Bayesian network can adjust other factors affected by it in time. (2) The Bayesian network can handle the overall risk state when certain risk factors remain the same in different stages of the same scene or in different scenes with the same nature. (3) The Bayesian network has a low requirement on the known information of the evaluation object, which can be used for reasoning in the case of incomplete and uncertain data. By combining expert experience with sample data, key points of contact between information can be captured, and major contradictions highlighted.

The Bayesian network has been widely used in the safety and security field because of intuitive appeal with available software. In terms of urban security, Tang et al. established a Bayesian network to analyze the risk of an urban dirty bomb attack [27]. Wu et al. established a comprehensive model based on the Bayesian network (BN) and the Delphi method for the rapid and dynamic assessment of the fire evolution process and consequences, in underground subway stations [28]. In terms of natural disasters, Han et al. proposed an earthquake disaster chain risk evaluation method that couples the Bayesian network and Newmark model based on natural hazard risk formation theory with the aim of identifying the influence of earthquake disaster chains [29]. In addition, the Bayesian network is also used in rural security, accident severity analysis, aviation safety, protection systems, etc. [30,31,32,33]. The literature above shows that the Bayesian network works well in solving uncertainty problems.

This paper aims to analyze the factors affecting the probabilities and consequences of gas leaks using Bayesian networks based on expert experience, Dempster–Shafer theory, field investigations, and case studies. A risk assessment model is established based on a Bayesian network to quantitatively assess the risks of gas leakage in school laboratories. The result can be used to guide the establishment of a toxic gas leakage warning system, which helps improve the safety management level of gas in the school laboratory, and reducing the possibility of gas leakage posing a threat to personal safety.

## 2. Methods

### 2.1. Bayesian Network

The Bayesian network, also known as the belief network, is an uncertainty processing model to analyze probabilities with insufficient data. The network topology of the Bayesian network is a directed acyclic graph (DAG) that contains a set of nodes, arcs, and conditional probability tables (CPTs). The nodes in the BN represent random variables ${\{X}_{1}{,X}_{2},\cdots {,X}_{n}\}$, the arcs define the dependencies between nodes, and the conditional probability tables reflect the probability distribution between node variables. The nodes in the BN can be divided into two types: the parent nodes and the child nodes. If there is an arc from node $X_{i}$ to another node $X_{j}$, then $X_{i}$ is called the parent of $X_{j}$ and $X_{j}$ is a child of $X_{i}$. The nodes in the BN can also be divided into root node, intermediate node, and leaf node. The leaf node has no child nodes, the root node has no parent nodes, and the other nodes are intermediate nodes. In a BN, the joint probability distribution can be easily calculated:

(1) $$P(X)=P(X_{1},X_{2},X_{3},\cdots ,X_{n})=\prod_{i=1}^{n}P(X_{i}|Pa(X_{i}))$$

where $P(X_{1},X_{2},X_{3},\cdots ,X_{n})$ represents the joint probability of the child node and $P(X_{i}|Pa(X_{i}))$ represents the conditional probability of each parent node of this node. Moreover, when new evidence Y appears, the probability of each node in the Bayesian network is updated dynamically:

(2) $$P(X|Y)=\frac{P(X)P(Y|X)}{P(Y)}=\frac{P(X)P(Y|X)}{\sum_{i=1}^{n}P(Y|X_{i})}$$

where P(X|Y) represents the conditional probability of X, P(Y) is the probability of evidence Y, P(X) is the prior probability of X, and $\sum_{i=1}^{n}P(Y|X_{i})$ represents the joint probability of evidence Y.

### 2.2. Conversion from BT to BN

In this study, the structure of the Bayesian network for assessing the risk of gas leakage in school laboratories was converted from a bow-tie diagram. The conversion was based on the work of Khakzad et al. [34]. The bow-tie diagram is a graphical model that shows the logic of event evolution, with the fault tree on the left and the event tree on the right. The fault tree is a pre-control process to identify the hazards that may lead to accidents (top event). While the event tree is a post-control process to show the possible consequences of unsafe events. The bow-tie diagram helps to understand which possible combination of primary events (hazards) will lead to the top event in the fault tree and which safety barrier failures will escalate the top event to a particular consequence in the event tree.

**Figure 1:**
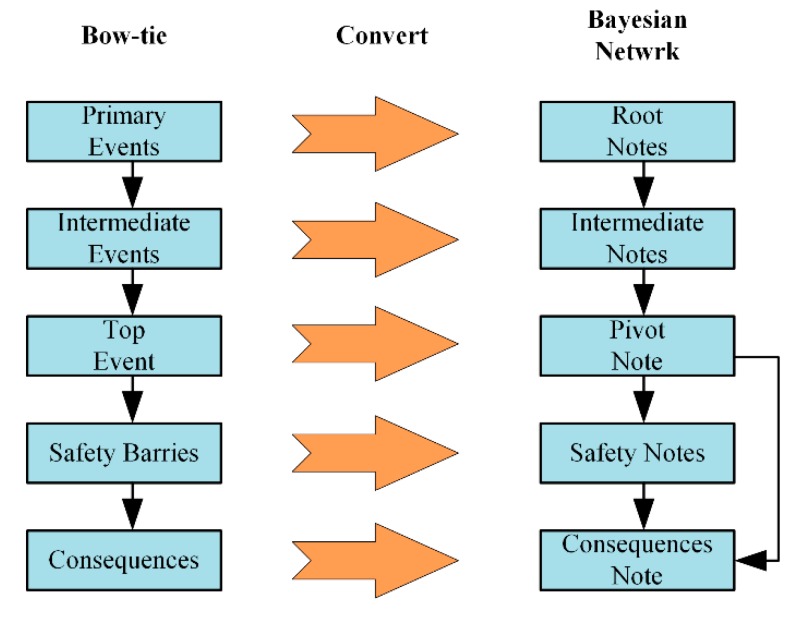
The mapping algorithm from the BT to Bayesian network (BN).

Conversion from the fault tree and the event tree to Bayesian networks includes a graphical and digital translation. In this study, the transformation of the graph is mainly considered. In the process of transforming the fault tree into a Bayesian network, the primary events, intermediate events, and the top event of the fault tree are expressed as root nodes, intermediate nodes, and leaf nodes in the equivalent Bayesian network. Similarly, each safety barrier of the event tree is represented by a safety node with two states, and the number of consequence nodes with as many consequences as the event tree is also added to the Bayesian network. The principle of converting a bow-tie graph into a Bayesian network is shown in Figure 1. The first step was to convert the elements in the BT into corresponding Bayesian nodes. When the estimated accident scenario is more complicated, some nodes need to be modified to establish a more reasonable model. The second step was to connect the BN nodes based on the causal relationship in the BT. Finally, the CPTs of each node was determined. In this study, the probability distribution of each node was determined by expert judgment and the Dempster–Shafer evidence theory.

### 2.3. Dempster–Shafer Theory

The Dempster–Shafer evidence theory was proposed by Dempster and developed by Deutsch, Yager, and Liu [35,36] as an imprecise reasoning theory, which has been widely used to deal with uncertain information. The theory is also applicable to information fusion, expert systems, intelligence analysis, multi-attribute decision analysis, and other fields.

The frame of discernment and the mass function are defined in Dempster–Shafer evidence theory. A complete set of basic incompatible propositions is called the frame of discernment. Let U be the frame of discernment, then the mass function needs to meet the following conditions:

(3) $$\{\begin{array}{c} m(\emptyset )=0\\ \sum_{A\subset U}m(A)=1 \end{array}$$

where m(A) is the mass function of event A, indicating the degree of trust to A, and is also the basic probability function of discernment U. The theory gives a synthesis rule for multi-source information, which combines basic reliability assignments from multiple sources to obtain a new reliability assignment. The synthesis rule is shown in Formula (4).

(4) $$(m_{1}⊕m_{2}⊕\cdots ⊕m_{n})(A)=\frac{1}{K}\sum_{A_{1}\cap A_{2}\cap \cdots A_{n}=A}m_{1}(A_{1})•m_{2}(A_{2})\cdots m_{n}(A_{n})$$

where $m_{1}$, $m_{2}$, …, $m_{n}$ are the basic probability functions of discernment U, where K is called the normalization factor, and 1−K reflects the conflict degree among $m_{1}$, $m_{2}$, …, $m_{n}$

(5) $$\begin{array}{l} K=\sum_{A_{1}\cap A_{2}\cap \cdots A_{n}\neq \emptyset }m_{1}(A_{1})•m_{2}(A_{2})\cdots m_{n}(A_{n})\\ =1-\sum_{A_{1}\cap A_{2}\cap \cdots A_{n}=\emptyset }m_{1}(A_{1})•m_{2}(A_{2})\cdots m_{n}(A_{n}) \end{array}$$

### 2.4. Bayesian Network Building

#### 2.4.1. Bayesian Nodes

**Figure 2:**
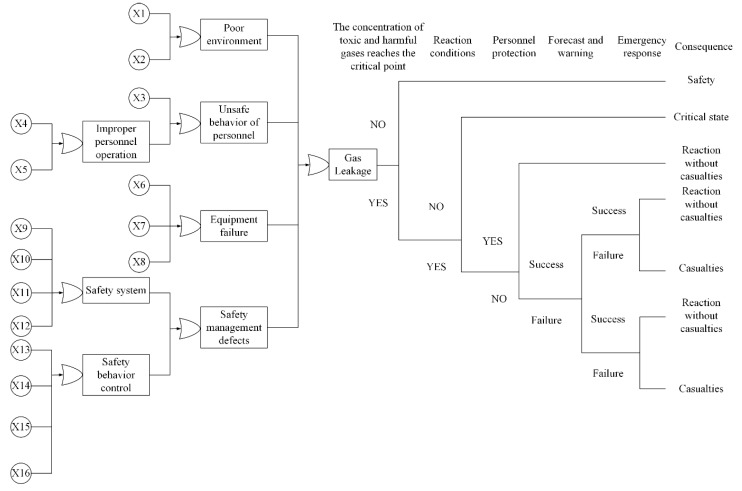
BT diagram for gas leakage in school laboratories.

The causes of gas leaks in school laboratories are complex and coherent, so it is difficult to construct the Bayesian network directly, and the bow-tie method can integrate and represent initial events, intermediate events, central event, safety measures, consequences, and causal relationships into one diagram, and then build the Bayesian network based on the constructed bow-tie diagram. In this study, the establishment of the bow-tie diagram was based on multiple gas leaks statistical data, related researches, and expert opinions. In the established bow-tie diagram, gas leakage is the central event, as shown in Figure 2. Table 1 provides the content represented by each symbol.

**Figure 3:**
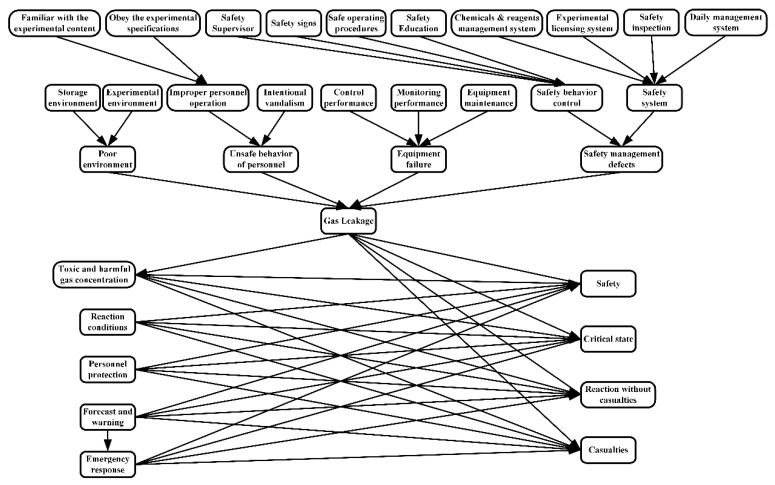
**The** Bayesian network of gas leakage in school laboratories.

According to the convert rule, a BN consists of 33 nodes (21 root nodes and 12 intermediate nodes) for representing gas leakage in school laboratories was established, as shown in Figure 3. The gas in this research is not only natural gas but can be any kind of flammable gases, such as methane, hydrogen, silane, formaldehyde, etc. By modifying the conditional probabilities, the method can be applied to different gases. The states of each node are listed in Table A1, and detailed descriptions of each Bayesian node are listed as follows:

1. Gas leakage: This node indicates that gas in the laboratory should not leak out of the device.
2. Poor environment: This node indicates that there are problems in the environment of the laboratory, mainly the experimental environment and the storage environment.
3. Unsafe behavior of personnel: In the laboratory, there are unsafe behaviors or operations during the experiment or routine work.
4. Equipment failure: Equipment related to the experiment or storage fails due to overload, improper use, improper maintenance, etc.
5. Security management defect: This node indicates that there are problems in the security management of the laboratory.
6. Storage environment: The storage of items should take into account such areas as environment, equipment, mode, location, temperature, ventilation, etc. The storage of various items should comply with relevant regulations.
7. Experimental environment: The layout, ventilation, lighting, test bench, and other factors of the experimental area have an impact on the safety of the experiment. Ventilation equipment commonly used in laboratories mainly includes fume hoods, atomic absorption hoods, or various exhaust hoods.
8. Improper personnel operation: This node indicates that the experimenter has improper operation or behavior during the experiment.
9. Intentional vandalism: Personnel in the laboratory deliberately make unsafe actions due to some unsafe thoughts, such as play, revenge, etc.
10. Control performance: The material structure, storage capacity, and airtightness of gas cylinders, gas pipes, reactors, etc., ensure the safety of storage and use of gases.
11. Monitoring performance: Overloading is a common cause of cylinder damage, and instruments, such as pressure gauges and thermometers, can reflect the safety status of cylinders.
12. Equipment maintenance: Equipment management personnel should be familiar with the laboratory equipment and have knowledge of the maintenance of equipment. When there is a serious problem with the equipment, the manager should look for a professional to perform maintenance and repair. Proper maintenance can reduce the probability of equipment failure.
13. Safety behavior control: This node represents constraints on the behavior of personnel in the laboratory, including safety supervision, safety education, warning slogans, etc.
14. Safety system: The safety management of laboratories should have a corresponding management system. The management and access to drugs, and whether experiments can be carried out should follow the established system.
15. Familiar with the experimental content: The experimental personnel should be prepared before the experiment, including mastering the nature of the drug and experimental methods, understanding the experimental process, be familiar with the characteristics and operation methods of the relevant instruments.
16. Obey the experimental specifications: The experimenter should follow the experimental specifications during the experiment and finishing stages. The experimenter cannot switch to other experiments or leave without permission.
17. Safety supervisor: This node indicates whether there is a special safety supervisor to supervise the behavior of personnel in the laboratory.
18. Safety signs: Areas in the laboratory and equipment for storing dangerous goods should have obvious signs, which have a warning effect on the personnel in the laboratory.
19. Safe operating procedures: The laboratory should have corresponding operating procedures to constrain the preparation before the experiment, the operation in the experiment, and the finishing after the experiment.
20. Safety education: Safety education and training should be carried out regularly in the laboratory to enhance the safety awareness of personnel.
21. Chemicals and reagents management system: The chemicals and reagents purchased and stored in the laboratory should be managed by a professional, as should the exhaust gas, etc.
22. Experimental licensing system: The experimenter can only conduct experiments after being approved by the safety controller.
23. Safety inspection: Regularly inspect the experimental environment, storage environment, and equipment to find potential safety hazards in time.
24. Daily management system: The laboratory should have daily management, such as checking doors and windows, cleaning, etc., to maintain the normal operation of the laboratory.
25. Toxic and harmful gas concentration: This node indicates the concentration of toxic and harmful gases in various areas of the laboratory.
26. Reaction conditions: This node indicates the conditions in the laboratory that cause the leaked gas to react, such as temperature, humidity, oxygen concentration, etc.
27. Personnel protection: The experimental personnel should take appropriate protective measures before the experiment. If an accident occurs, these protective measures can effectively protect the experimental personnel and mitigate the harm of the experimenters.
28. Forecast and warning: This node indicates whether the detector can emit an audible and visual alarm or whether the laboratory personnel can detect and issue an alarm in time when the accident happens.
29. Emergency response: This node indicates whether personnel in the laboratory can take appropriate emergency measures or evacuate in time when the accident happens.
30. Safety: This node indicates that there is no possibility of economic losses and casualties in the laboratory.
31. Critical state: This node indicates that the toxic and harmful gases in the laboratory reached the threshold value of the reaction but were not triggered.
32. Reaction without casualties: The leaked gas reacted without causing casualties.
33. Casualties: There are casualties in the accident in the laboratory.

#### 2.4.2. Conditional Probability Tables

To analyze the evolution of gas leaks in school laboratories, we need to get the probability distribution and dependency intensity of Bayesian nodes, which are represented by the conditional probability table of each node. In general, the probability distribution of Bayesian nodes is obtained by a parameter learning method or an expert scoring method. The method of parameter learning requires massive data, of which there is not enough on laboratory gas leakage accidents, so it is difficult to determine CPTs by parameter learning. In this study, we invited four experts with rich experience in laboratory safety management to fill out CPT questionnaires of the proposed Bayesian network. The CPTs of each node were obtained according to expert experience, with consistent processing using the Dempster–Shafer evidence theory. Then, we used the Dempster–Shafer evidence theory to analyze the data of each expert and determine the probability distribution of each node.

Let us take the determination of the CPT for the “Toxic and harmful gas concentration” node as an example. The probability distribution of “Toxic and harmful gas concentration” depends on the state of “Gas Leakage”. As shown in Table 2, $m_{1}(1,2),\ldots ,m_{4}(1,2)$ represent the probability distributions given by four experts, where $m_{4}(1,2)$ with the value (0.61, 0.39) means that the fourth expert thought the probability of “Toxic and harmful gas concentration” reaching the critical point of reaction is 0.61 on the premise that the state of “Gas Leakage” is “Yes”, and the probability of “the critical value is not reached” is 0.39. Based on Equations (4) and (5), the probability distribution of “Toxic and harmful gas concentration” is calculated as follows:

*K* = *m*_1_(*A*_1_)**·***m*_2_(*A*_1_)**·***m*_3_(*A*_1_)·*m*_4_(*A*_1_) + *m*_1_(*A*_2_)·*m*_2_(*A*_2_)·*m*_3_(*A*_2_)·*m*_4_(*A*_2_) = 0.11475 *m*(*A*_1_) = (1/*K*)·*m*_1_(*A*_1_)·*m*_2_(*A*_1_)·*m*_3_(*A*_1_)·*m*_4_(*A*_1_) = 0.558

Just like what has been presented above, we used Dempster–Shafer evidence theory to deal with the judgment data from different experts to determine the probability distribution of all nodes.

**Figure A1:**
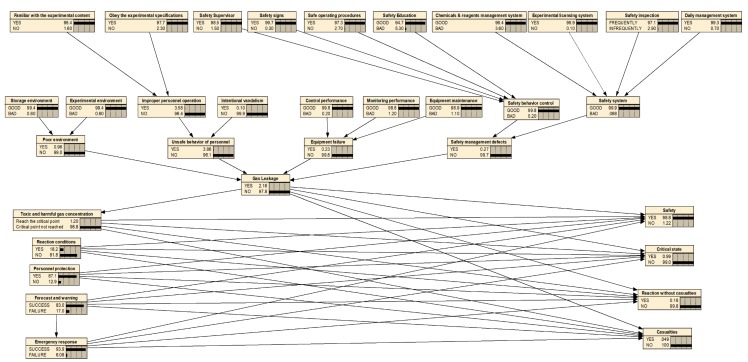
Initial BN with conditional probability tables (CPTs).

Through the above method, an initial Bayesian network with probability distribution can be obtained. As shown in Figure A1, the proposed Bayesian network can be used to analyze the evolution of gas leaks in school laboratories. In this study, the Bayesian network probability inference was conducted using Netica (Netica 4.16, Norsys Software Corp., Vancouver, BC, Canada), which has been widely used in Bayesian network analyses.

## 3. Results and Discussion

In this study, the Bayesian network can also perform predictive analysis by obtaining the state of certain root nodes for a given accident scenario. Through predictive analysis, we can quantitatively simulate the different states after gas leakage and the consequences of the accident scenario. Meanwhile, the main factors affecting the probability of gas leakage in laboratories were examined based on sensitivity analysis, assessing the impacts of different factors on the consequences of accidents.

### 3.1. Critical Threats Identification and Analysis

Sensitivity analysis refers to an uncertainty analysis technology that identifies sensitive factors that have a significant impact on the object from several influential factors. The analysis software Netica was used in this study to achieve the sensitivity analysis function, which can be used to analyze the influence of various factors on “Gas Leakage” and “Casualties”. In Netica, click the node you want to analyze, such as “Gas Leakage”, and select “Sensitivity to findings” in “Network” to generate a sensitivity analysis report.

The sensitivity analysis results of other nodes affecting “Gas Leakage” are listed in Table 3. As can be seen from the data in the table, the node “Gas Leakage” was mainly affected by “Unsafe behavior of personnel”, which conformed to an actual situation, since, according to accident cases, most accidents are caused by improper behavior of personnel. Therefore, to reduce the possibility of gas leakage, personnel misconduct should be avoided as much as possible.

The sensitivity analysis results of the node “Unsafe behavior of personnel” to the root nodes are listed in Table 4. As shown above, the node “Unsafe behavior of personnel” is mainly affected by “Obey the experimental specifications” and “Familiar with the experimental content”. Reducing the probability of “Unsafe behavior of personnel” requires attention to these two aspects. Before the experiment begins, it should be ensured that the relevant personnel of the experiment have mastered the relevant content and specifications of the experiment, and implemented the safety responsibility system of the laboratory. The safety supervisor should supervise the behavior and operation of the experimenter throughout the process to avoid the occurrence of unsafe behavior.

In addition, the data in Table 3 shows that the sensitivity of the node “Gas Leakage” to “Safety management defect” is small, and this phenomenon indicates that the probability of gas leakage directly caused by “Safety management defect” is low. Although the immediate cause of gas leakage is mainly human, environmental, and equipment problems, it is often accompanied by safety management defects. The scenario combinations of these four nodes and the probability of gas leakage are shown in Table 5. As can be seen from the data in the table, the probability of gas leakage increases rapidly when there is a problem with people, the environment, or equipment and is accompanied by safety management defects.

### 3.2. Impacts of Different Factors on the Consequences

**Figure 4:**
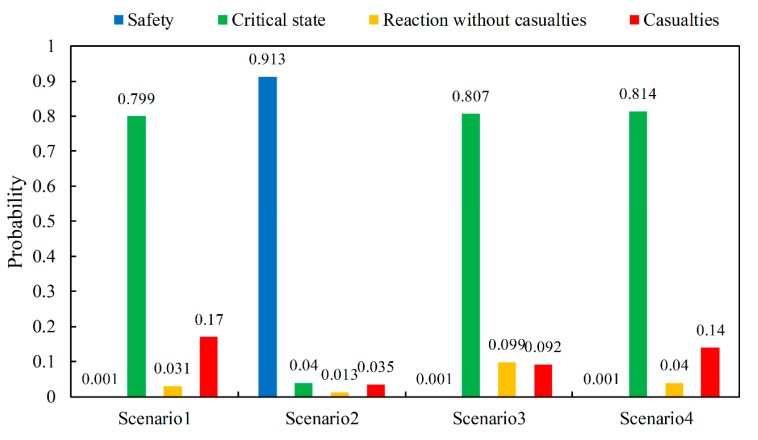
Inference results of consequences on the condition of different scenarios.

To analyze the impact of each safety node on the consequences of the accident, the sensitivity analysis of the node “Casualties” is carried out. The results are shown in Table 6. As can be seen from the data in the table, in addition to the node “Gas Leakage”, the node “Casualties” is mainly affected by “Toxic and harmful gas concentration”. Among these nodes, the node “Reaction conditions” is limited by objective factors, such as the state of the laboratory, which is difficult to control by humans, and the node “Emergency response” is affected by the node “Forecast and warning”. Therefore, this part mainly discusses the impact of toxic and harmful gas concentrations, personnel protection, and forecasting and warning on the consequences of accidents. The combination of several states of these nodes are given as listed in Table 7, and the estimated probability of consequences is shown in Figure 4.

As shown in Figure 4, when “Toxic and harmful gas concentration” transfers from “Critical point not reached” to “Reach the critical point”, the probability of “Yes” of “Safety” drops from 0.913 to 0.001, the probability that the “Critical state” is in the state “Yes” increases from 0.04 to 0.799, and the probability of “Yes” of “Casualties” increases from 0.035 to 0.17. The results show that the concentration of toxic and harmful gases in the laboratory has a great impact on the consequences of the accident and should be controlled properly. Therefore, in the daily operation of the laboratory, the ventilation state should be well maintained to reduce the probability that the concentration of toxic and harmful gases reaches the critical point when the gas leaks, and mitigate the consequences of the accident.

Similarly, as can be seen from Figure 4, when “Personal protection” transfers from “Yes” to “No”, the probability of “Yes” of “Casualties” is significantly increased. Personal protective measures can protect the user, reduce the probability of potential damage to the human body caused by the reaction. In addition, “Forecast and warning” can also reduce the severity of the consequences of the accident. After successful “Forecasting and warning”, personnel in the laboratory can take emergency measures to control the leaked parts in time, and also remind relevant personnel to evacuate quickly to reduce the probability of casualties.

### 3.3. Validity of Risk Assessment

Quantitative risk analysis differs from other areas of applied science as it attempts to model events that are unlikely to occur, so the validity of the risk assessment model needs to be verified. Borg et al. summarized two major risk interpretations and made recommendations for the validity of risk assessments based on different risk interpretations [37]. Aven explained the general definitions of different types of validity in more detail and summarized the scope and methods of different validity definitions [38].

The effectiveness of the risk assessment is defined in the following categories:

The degree to which the produced risk numbers are accurate compared to the true underlying risk (V1).

The degree to which the assigned probabilities adequately describe the assessor’s uncertainties of the unknown quantities considered (V2).

The degree to which the epistemic uncertainty assessments are complete (V3).

The degree to which the analysis addresses the right quantities (V4).

(V1) and (V4) are suitable for classical methods, and (V2), (V3), and (V4) are suitable for Bayesian prediction methods. Analysis of the methods and contents of this study shows that (V2) is suitable for verifying the validity of the risk assessment in this study. It is not straightforward to verify that the validity requirement (V2) is met. Some important principles and procedures are involved, as follows.

(i) Coherent uncertainty assessments are achieved by using the rules of probability, including Bayes’ theorem, for updating of assessments in the case of new information.

(ii) Comparisons are made with relevant observed relative frequencies if available.

(iii) Training in probability assignments is required to make assessors aware of heuristics as well as other problems of quantifying probabilities, such as superficiality and imprecision.

(iv) Using models, including probability models, to simplify the assignment process.

(v) Using procedures for incorporating expert judgments.

(vi) Accountability: The basis for all probability assignments must be identified.

These principles and procedures provide a basis for establishing a standard for the probability assignments; the aim being to extract (elicit) and summaries knowledge about the unknown quantities (parameters), using models, observed data, and expert opinions. It seems reasonable to say that the requirement (V2) is met provided that this standard is followed.

In this study, the risk assessment model is based on the Bayesian network and satisfies condition (i). Since there are no relevant observed relative frequencies, but a large number of case studies were provided to the experts. With the help of experts’ background knowledge, the error between subjective data and objective data can be reduced to meet conditions (iii) and (vi). Expert experience was obtained through a questionnaire. After obtaining the scores of the experts, the Dempster–Shafer evidence theory was used to process the data from different experts, and finally, the probability distribution was obtained, so the conditions (iv) and (v) are met. In addition, the research had no interest or conflict with the selected experts, ensuring the motivation of the experts. In summary, in this study, the above criteria were followed in the process of a probability distribution, (V2) was met, and the validity of risk assessment was verified.

### 3.4. Accident Scenario Predictive Analysis

**Figure A2:**
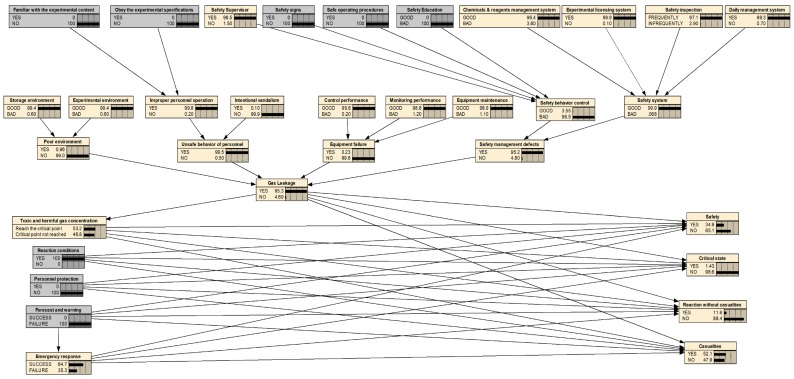
A real-world accident scenario modeling.

In this study, the model was demonstrated as an accident scenario through the “7.3” incident at Zhejiang University. In the accident, two teachers mistakenly passed carbon monoxide gas to another laboratory, caused one fatality. According to the accident investigation, some root nodes with certain states are shaded in gray in Figure A2. According to the accident cause investigation, “Familiar with the experimental content” and “Obey the experimental specifications” were assigned the “No” state, “Safety education” was assigned the “Bad” state, and “Safe operation procedures” and “Drug management system” were assigned the “No” state. According to the serious consequences, “Reaction condition” was assigned the “Yes” state, “Personnel protection” was assigned the “No” state, and “Forecast and warning” was assigned the “Failure “state. As shown in Figure A2, the occurrence probability of gas leakage was 95.3%. “Casualties” had the highest probability of accident consequence at 52.1%. The accident scenario shows that the predictive results of this model are consistent with reality.

## 4. Conclusions

To comprehensively represent and assess the risk of gas leakage in school laboratories, this study applied an integrated risk assessment model for rapid and dynamic modeling gas leaks in school laboratories based on the Bayesian network. The model was used to analyze how the environment, personnel behavior, equipment, and safety management affect the probability of gas leakage and the effects of toxic and harmful gas concentrations on the consequences of the accident. The main conclusions are:

(1) The behavior of personnel has a significant impact on the probability of gas leakage. If there are unsafe behaviors of personnel, the probability of gas leakage would still reach 39.3%, even though the environment, equipment, and management are in good condition. In terms of personnel behavior, it is necessary to pay attention to the experimenter’s compliance with the experimental specifications.

(2) The probability of gas leakage directly caused by safety management defects is extremely low, but problems with people, environment, or equipment are often accompanied by safety management defects, which increase the probability of gas leakage.

(3) The concentration of toxic and harmful gases has the greatest impact on the consequences of accidents. The ventilation state of the laboratory should be controlled strictly to reduce the probability of toxic and harmful gases reaching the critical point.

(4) Effective personnel protection, successful forecasting, and early warning can effectively mitigate the consequences of accidents.

With the lack of accident data in laboratories, the probability distribution of Bayesian nodes was obtained based on expert experience, but it could present comparatively quantitative risk distribution for reference. In addition, the model can provide guidance for risk analysis of other accidents related to laboratory safety, such as explosions and leakage of liquid reagents, by simply adjusting some nodes, node relationships, and conditional probabilities in the model. However, the model has little guiding effect on the risk analysis of safety issues with strong human initiative, such as suicide. Due to the convenience of the probability update of this model, with the constructed network, quantitative risk assessment can be performed quickly before each experiment to assist the safety management and decrease losses. In future work, with real-time monitoring data, such as toxic and harmful gas concentrations, this model can be dynamically mobilized to achieve real-time dynamic quantitative risk assessment and early warning of gas leakage in the laboratory to support the emergency decision and treatment.

## Figures and Tables

**Table 1:** Instruction of primary BT events.

Symbol	Description
X1	Storage environment
X2	Experimental environment
X3	Familiar with the experimental content
X4	Obey the experimental specifications
X5	Intentional vandalism
X6	Control performance
X7	Monitoring performance
X8	Equipment maintenance
X9	Safety supervisor
X10	Safety signs
X11	Safe operating procedures
X12	Safety education
X13	Chemicals and reagents management system
X14	Experimental licensing system
X15	Safety inspection
X16	Daily management system

**Table 2:** Experts’ judgmental data and the final condition probabilities of the node.

Causal Relationship Element	Experts’ Judgment				Calculated Results
**Gas Leakage**	***m*_1_(1,2)**	***m*_2_(1,2)**	***m*_3_(1,2)**	***m*_4_(1,2)**	Toxic and harmful gas concentration
**Yes**	(0.5, 0.5)	(0.6, 0.4)	(0.35, 0.65)	(0.61, 0.39)	(0.558, 0.442)
**No**	(0.01, 0.99)	(0.02, 0.98)	(0.1, 0.9)	(0.42, 0.58)	(0, 1)

**Table 3:** Sensitivity analysis of “Gas Leakage”.

Node	Mutual Info	Percent	Variance of Beliefs
Gas Leakage	0.15001	100	0.0210947
Unsafe behavior of personnel	0.05893	39.3	0.0057192
Equipment failure	0.00051	0.338	0.0000326
Poor environment	0.00034	0.23	0.0000149
Safety management defect	0.00004	0.0279	0.0000016

**Table 4:** Sensitivity analysis of “Unsafe behavior of personnel”.

Node	Mutual Info	Percent	Variance of Beliefs
Unsafe behavior of personnel	0.23588	100	0.0371195
Improper personnel operation	0.20644	87.5	0.0341315
Obey the experimental specifications	0.11076	47	0.0202325
Familiar with the experimental content	0.05893	25.5	0.0111796
Intentional vandalism	0.00470	1.99	0.0009233

**Table 5:** Analysis of the impact of “Safety management defect” on “Gas Leakage”.

Scenarios	Poor Environment	Unsafe Behavior of Personnel	Equipment Failure	Safety Management Defect	Probability of Gas Leakage
Scenario1	Yes	No	No	No	2.40%
Scenario2	Yes	No	No	Yes	34.40%
Scenario3	No	Yes	No	No	39.10%
Scenario4	No	Yes	No	Yes	98.60%
Scenario5	No	No	Yes	No	9.70%
Scenario6	No	No	Yes	Yes	92.50%

**Table 6:** Sensitivity analysis of “Casualties”.

Node	Mutual Info	Percent	Variance of Beliefs
Casualties	0.00608	100	0.0004885
Gas Leakage	0.00271	44.6	0.0000108
Toxic and harmful gas concentration	0.0026	43.7	0.0000164
Reaction conditions	0.00099	16.2	0.0000009
Personnel protection	0.00027	4.36	0.0000003
Emergency response	0.00025	4.1	0.0000003
Forecast and warning	0.00022	3.55	0.0000002

**Table 7:** Initial settings for some Bayesian network (BN) nodes to evaluate the impact of these nodes on consequences.

Bayesian Nodes	Setup of Bayesian Nodes			
	Scenario1	Scenario2	Scenario3	Scenario4
Toxic and harmful gas concentration	Reach the critical point	Critical point not reached	Reach the critical point	Reach the critical point
Personnel protection	No	No	Yes	No
Forecast and warning	Failure	Failure	Failure	Success

## Appendix A

States of Bayesian nodes and the Bayesian network generated by Netica.

**Table A1:** States of Bayesian nodes.

Nodes	State of Nodes
Gas Leakage	① Yes ② No
Poor environment	① Yes ② No
Unsafe behavior of personnel	① Yes ② No
Equipment failure	① Yes ② No
Safety management defects	① Yes ② No
Storage environment	① Good ② Bad
Experimental environment	① Good ② Bad
Improper personnel operation	① Yes ② No
Intentional vandalism	① Yes ② No
Control performance	① Good ② Bad
Monitoring performance	① Good ② Bad
Equipment maintenance	① Good ② Bad
Safety behavior control	① Good ② Bad
Safety system	① Good ② Bad
Familiar with the experimental content	① Yes ② No
Obey the experimental specifications	① Yes ② No
Safety supervisor	① Yes ② No
Safety signs	① Yes ② No
Safe operating procedures	① Yes ② No
Safety education	① Good ② Bad
Chemicals and reagents management system	① Good ② Bad
Experimental licensing system	① Yes ② No
Safety inspection	① Frequently ② Infrequently
Daily management system	① Yes ② No
Toxic and harmful gas concentration	① Reach the critical point ② Critical point not reached
Reaction conditions	① Yes ② No
Personnel protection	① Yes ② No
Forecast and warning	① Success ② Failure
Emergency response	① Success ② Failure
Safety	① Yes ② No
Critical state	① Yes ② No
Reaction without casualties	① Yes ② No
Casualties	① Yes ② No

## References

[1] Burnett L.A.C. (2009). Risk Assessment in the Research Laboratory.

[2] Foster B.L. (2004). Laboratory safety program assessment in academia. Chem. Health Saf..

[3] Foster B.L. (2005). The Chemical Inventory Management System in academia. Chem. Health Saf..

[4] Shariff A.M., Norazahar N. (2012). At-risk behaviour analysis and improvement study in an academic laboratory. Saf. Sci..

[5] Si H., Ji H., Zeng X. (2012). Quantitative risk assessment model of hazardous chemicals leakage and application. Saf. Sci..

[6] Tsung-Chih W., Chi-Wei L., Mu-Chen L. (2007). Safety climate in university and college laboratories: Impact of organizational and individual factors. J. Saf. Res..

[7] Zhang C., Wu J., Huang C., Jiang B. (2018). A model for the representation of emergency cases. Nat. Hazards.

[8] Leggett D.J. (2012). Identifying hazards in the chemical research laboratory. Process Saf. Prog..

[9] Marendaz J.L., Suard J.C., Meyer T. (2013). A systematic tool for Assessment and Classification of Hazards in Laboratories (ACHiL). Saf. Sci..

[10] Mulcahy M.B., Boylan C., Sigmann S., Stuart R. (2017). Using bowtie methodology to support laboratory hazard identification, risk management, and incident analysis. J. Chem. Health Saf..

[11] Omidvari M., Mansouri N., Nouri J. (2015). A pattern of fire risk assessment and emergency management in educational center laboratories. Saf. Sci..

[12] Jaeger C.D. (2002). Vulnerability Assessment Methodology for Chemical Facilities (VAM-CF). Chem. Health Saf..

[13] Lemley J.R., Fthenakis V.M., Moskowitz P.D. (2010). Security risk analysis for chemical process facilities. Process Saf. Prog..

[14] Leggett D.J. (2012). Lab-HIRA: Hazard identification and risk analysis for the chemical research laboratory: Part 1. Preliminary hazard evaluation. J. Chem. Health Saf..

[15] Leggett D.J. (2012). Lab-HIRA: Hazard identification and risk analysis for the chemical research laboratory. Part 2. Risk analysis of laboratory operations. J. Chem. Health Saf..

[16] Ouédraogo A., Groso A., Meyer T. (2011). Risk analysis in research environment-Part I: Modeling Lab Criticity Index using Improved Risk Priority Number. Saf. Sci..

[17] Ouédraogo A., Groso A., Meyer T. (2011). Risk analysis in research environment-Part II: Weighting Lab Criticity Index using the Analytic Hierarchy Process. Saf. Sci..

[18] Foster R.W., Robertson C.S. (2001). Monitoring indoor air quality in the laboratory building. Chem. Health Saf..

[19] Hui S., Duan G. (2012). Risk Quantitative Calculation and ALOHA Simulation on the Leakage Accident of Natural Gas Power Plant. Procedia Eng..

[20] Li X., Chen G., Zhu H. (2016). Quantitative risk analysis on leakage failure of submarine oil and gas pipelines using Bayesian network. Process Saf. Environ. Prot..

[21] Murvay P.S., Silea I. (2012). A survey on gas leak detection and localization techniques. J. Loss Prev. Process Ind..

[22] Warzyniec E. (2000). Safe handling of compressed gases and cryogenic liquids. Chem. Health Saf..

[23] Aven T.J.R.E., Safety S. (2012). The risk concept—historical and recent development trends. Reliab. Eng. Syst. Saf..

[24] Aven T., Krohn B.S. (2014). A new perspective on how to understand, assess and manage risk and the unforeseen. Reliab. Eng. Syst. Saf..

[25] Aven T., Safety S. (2010). On how to define, understand and describe risk. Reliab. Eng. Syst. Saf..

[26] Leitch M. (2010). ISO 31000: 2009—The new international standard on risk management. Risk Anal..

[27] Tang Z., Li Y., Hu X., Wu H. (2019). Risk Analysis of Urban Dirty Bomb Attacking Based on Bayesian Network. Sustainability.

[28] Wu J., Hu Z., Chen J., Li Z. (2018). Risk Assessment of Underground Subway Stations to Fire Disasters Using Bayesian Network. Sustainability.

[29] Han L., Ma Q., Zhang F., Zhang Y., Zhang J., Bao Y., Zhao J. (2019). Risk assessment of an earthquake-collapse-landslide disaster chain by Bayesian network and Newmark models. Int. J. Environ. Res. Public Health.

[30] Tang T., Zhu S., Guo Y., Zhou X., Cao Y. (2019). Evaluating the safety risk of rural roadsides using a Bayesian network method. Int. J. Environ. Res. Public Health.

[31] Zhu L., Lu L., Zhang W., Zhao Y., Song M. (2019). Analysis of Accident Severity for Curved Roadways Based on Bayesian Networks. Sustainability.

[32] Zhou T., Zhang J., Baasansuren D. (2018). A hybrid HFACS-BN model for analysis of Mongolian aviation professionals’ awareness of human factors related to aviation safety. Sustainability.

[33] Ghasemi F., Sari M., Yousefi V., Falsafi R., Tamošaitienė J. (2018). Project portfolio risk identification and analysis, considering project risk interactions and using Bayesian networks. Sustainability.

[34] Khakzad N., Khan F., Amyotte P. (2013). Dynamic safety analysis of process systems by mapping bow-tie into Bayesian network. Process Saf. Environ. Prot..

[35] Dempster A.P. (1967). Upper and Lower Probabilities Induced by a Multivalued Mapping. Ann. Math. Stat..

[36] Yager R.R. (2008). Classic Works of the Dempster-shafer Theory of Belief Functions. Stud. Fuzziness Soft Comput..

[37] Borg A., Njå O. (2013). The concept of validation in performance-based fire safety engineering. Saf. Sci..

[38] Aven T. (2011). Quantitative Risk Assessment: The Scientific Platform.

